# Reversal of anxiety-like depression induced by chronic corticosterone by crocin I and surface-enhanced Raman spectroscopy monitoring of plasma metabolites

**DOI:** 10.3389/fphar.2025.1540551

**Published:** 2025-02-27

**Authors:** Dandan Zhang, Zhuodi Wu, Doudou Yang, Guanjie Zhao, Yanru Zhang, Weifeng Mou, Yinku Liang

**Affiliations:** School of Biological Sciences and Engineering, Shaanxi University of Technology, Hanzhong, China

**Keywords:** crocin I, anxiolytic-like depression, pharmacokinetics, surface-enhanced Raman spectroscopy, trace monitoring

## Abstract

Anxiety disorders and depression often co-occur and lack broadly available treatments. Gardenia extract significantly associated with treatment of anxiety-like depression. Based on the dose effect hypothesis and previous studies, it is speculated that crocin I, the main component of gardenia, is significantly related to the treatment of anxiety-like depression. The present study aimed to verify the reversal effect of crocin I on chronic corticosterone-induced anxiety-like depression, and to further explore its metabolic process *in vivo*. Ultimately, a new method for rapid and sensitive detection of trace substances was established. In this study, the rat model of anxiety-like depression was induced by chronic corticosterone. The effects of crocin I were explored by combining behavioral, pathological sections and ELASA data. It is the first time that crocin I can reverse the morphological changes of hippocampus induced by corticosterone in rats. In terms of behavior, crocin Ⅰ can significantly improve the anxiety-like depressive behavior exhibited by model rats in water maze and sugar water preference experiments. It can also repair neuronal cell damage in the Dentate gyrus, CA1, and CA3 areas of the hippocampus. It also regulates the expression levels of monoamine neurotransmitters in the rat brain, thereby exerting an anti-anxiety-like depression effect. Pharmacokinetic analysis was performed to determine the metabolic process *in vivo*. Further integrating Surface-Enhanced Raman Scattering (SERS) technology, a highly sensitive and rapid detection method for trace substances had been established. It was first discovered that crocin I can reverse the changes in rat hippocampal morphology caused by corticosterone. It was determined that crocin Ⅰ can reverse the anxiety-like depression induced by chronic corticosterone and exert its therapeutic effect by regulating the levels of neurotransmitters in the brain. *In vivo* pharmacokinetic experiments revealed that crocin Ⅰ could not pass through the intestinal barrier into the blood, but its metabolite crocetin could pass through the intestinal barrier into the blood. Finally, by synthesizing silver nanoparticles, a detection method for trace amounts of the metabolite crocetin in blood samples was established for the first time.The calculated enhancement factor is 4.49 × 10^11^. The method was stable and reproducible over a week. This series of studies revealed the great potential of crocin I in treating comorbid anxiety and depression. It shortens the distance from theoretical research to clinical application.

## Graphical Abstract

**Figure:**
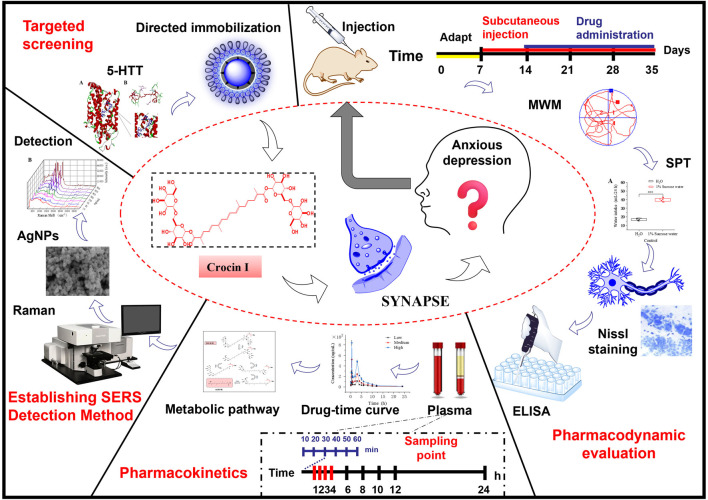

## Highlights

- It was first discovered that crocin I can reverse the changes in rat hippocampal morphology caused by corticosterone. It was determined that crocin I can reverse the anxiety-like depression induced by chronic corticosterone and exert its therapeutic effect by regulating the levels of neurotransmitters in the brain.
- It was found that crocin I itself makes it difficult to cross the intestinal barrier and enter the blood, but its metabolite crocetin can successfully pass through the blood circulation and be detected.
- For the first time, surface-enhanced Raman spectroscopy was established to detect trace amounts of crocetin in plasma.

## 1 Introduction

Anxiety disorders and depression are complex disorders that often occur together. There are currently no widespread treatments for this comorbidity (Gao et al., 2023). Traditional medicines have made important contributions in the treatment of anxiety-like depression (Jiang et al., 2024; Mao et al., 2024). However, due to the complex disease mechanism and long treatment cycle, long-term use can easily cause side effects such as drowsiness, weight gain, sexual dysfunction, liver and kidney damage, etc., which limits the wider application of traditional drugs (Xiong et al., 2024; Yerubandi et al., 2024). In the long-term use and practice of natural medicines, the safety and side effects of natural drugs have been continuously screened and verified. The toxic and side effects are relatively small, which is more conducive to long-term use by patients (Guo et al., 2024). Some natural medicines have the functions of regulating the function of the central nervous system, improving emotional state, reducing stress response, relieving stress, and improving sleep (Caldas et al., 2024; Chen et al., 2024; Gangwar et al., 2024). It can specifically relieve symptoms of anxiety and depression. Through scientific research, we can screen out anti-anxiety and anti-depressant drugs with proven efficacy and provide patients with more effective treatment options (Zhong et al., 2024). Therefore, finding natural small molecules that can target and regulate the anxiety and depression systems has become a hot topic in modern research (Alagapan et al., 2023; Han et al., 2023). A large number of studies have shown that changes in serotonin (5-HT) levels directly affect the development of anxiety and depression, playing a vital and irreplaceable role (Blumenthal et al., 2021; Kendrick and Collinson, 2022; Lee et al., 2023; Zhou et al., 2023). The serotonin transporter, as a high-affinity transporter of serotonin, can selectively inhibit the reuptake of serotonin by neurons. This can regulate the transduction of neural signals and exert a therapeutic effect (Bligh-Glover et al., 2000; Caspi et al., 2003; Zanardi et al., 2001).

Gardenia jasminoides Ellis is derived from the dried and mature fruit of Gardenia jasminoides Ellis of Rubiaceae. The chemical components that have been reported so far include iridoid glycosides, monoterpenes, diterpenes, triterpenes, flavonoids, organic acids and other compounds (Wang et al., 2024). Studies have shown that gardenia extract has a significant improvement effect on neurological diseases, including anxiety, depression, Alzheimer’s disease, and Parkinson’s disease. Crocin I is a diterpenoid compound of Gardenia jasminoides Ellis, which is one of its main active components (Hou et al., 2024). In the previous research work, a highly mature protein-oriented immobilized screening technology system was successfully established by using the drug target recognition mechanism (Liang et al., 2022; Tian et al., 2022; Wang et al., 2019; Zhao et al., 2022). The surface-enhanced Raman technology and affinity analysis method were innovatively combined. Targeted screening identified crocin I as a potential candidate compound for the treatment of anxiety-like depression (Zhang et al., 2023a). Despite preliminary results being obtained, the exact therapeutic efficacy and mechanism of action for treating anxiety-like depressive disorders remain unclear. Further exploration is still needed for its metabolism *in vivo* and effective detection methods. This situation highlights the urgency of further research, which aims to fully reveal the pharmacological properties of crocin I and provide a new scientific basis and potential drug candidates for the treatment of anxiety-like depression.

In this study, we established an animal model of anxiety-like depression induced by chronic corticosterone (Burokas et al., 2017; Du Preez et al., 2021; Moigneu et al., 2023; Zhang et al., 2023b). The anti-anxiety-like depression effect of crocin I was evaluated and the mechanism of action was clarified. Further, *in vivo* pharmacokinetics studies were conducted on crocin I to clarify its metabolic process. Then we innovated methods and established a rapid detection method for crocin I. The implementation of this program is expected to reveal the mechanism of action of Crocin I and fill the existing knowledge gaps. Provide potential candidate drugs and methodological references for the treatment of anxiety-like depression.

## 2 Materials and methods

All procedures were preapproved by the Center for Laboratory Animal Management and Welfare Ethics of Northwest University. Experimental procedures were conducted following the Code of Ethics for Laboratory Animal Welfare (Resolution No. NWU-AWC-20230503R). The reagents and materials used in the experiments are listed in Supplementary Tables S1, S2.

### 2.1 Anxiolytic-like depression pharmacodynamic evaluation

#### 2.1.1 Animals

Male Sprague Dawley (SD) rats, 4 weeks old (SPF grade), were obtained from the Experimental Animal Center, Department of Medicine, Xi’an Jiaotong University, China. The rats were placed in a 25 °C ± 1 environment for 12 h of light/dark cycle (8 a.m./8 p.m. light on/off) and allowed access to drink and chow freely.

#### 2.1.2 Protocol to establish corticosterone-induced anxiety-like depression

Anxiety-like depression rat model is induced by chronic induction of corticosterone. Five-week-old SD male rats (8/cage) randomly received 0.45% soluble Corticosterone suspension in *β*-cyclodextrin. The number of rats in each group has been supplemented in part 1.2 of the manuscript method. Specifically described as “The rats were randomly divided into 7 groups, 8 rats in each group, a total of 56 rats. The number of groups were model group, blank control group, positive drug group, low, middle, and high dose of Crocin I group, and gardenia extract group respectively. The model was established by subcutaneous injection of corticosterone (20 mg⋅kg^−1^) for 21 consecutive days. The rats in the positive drug group were gavaged with paroxetine hydrochloride (5 mg⋅kg^−1^) once a day for 21 consecutive days. Similarly, the low, middle, and high dose of the Crocin I group was intragastrically infused with 2.25, 4.5, and 9 mg⋅kg^−1^, respectively, and the extract of Gardenia jasminoides was infused with 0.9 g⋅kg^−1^. The control group and model group were given the same volume of normal saline. Solution preparation methods are described in Supplementary Material S1.1.

#### 2.1.3 Behavioral tests

After the model was established. Morris water maze tests were conducted to evaluate learning and memory abilities (Zheng et al., 2021). Depression was assessed by the preference for sweet foods in healthy rats (Tan et al., 2020) Details were provided in the Additional file: SI 1.2-1.3.

#### 2.1.4 Tissue sample collection and nissl staining

The rats were deeply anesthetized with 2% sodium pentobarbital. After 15 min, the rats heart were perfused and fixed with normal saline and 4% paraformaldehyde, respectively. Then the brain tissues of rats in different groups were collected. The brain tissue was cryosectioned at a thickness of 3 μm, and then Nissl staining was performed to analyze the hippocampal region of interest. The staining process is detailed in Supplementary Material S1.4.

#### 2.1.5 Determination of monoamine neurotransmitters

The contents of Serotonin transporter (5-HTT, JW.RA1057), dopamine (DA, JW.RA1621), *γ*-aminobutyric acid (GABA, JW.RA1389) and noradrenaline (NA, JW.RA2154) in the hippocampus were measured by ELISA kit. The tissue samples were added to PBS low-temperature homogenate according to 1:10, and the homogenate was centrifuged at 10,000 rpm at 4 °C (1,000 rpm) for 10 min. The supernatant was taken to detect the content of the sample.

### 2.2 Pharmacokinetic analysis

#### 2.2.1 Experimental grouping

Forty-eight rats were housed with the air conditioning set at 26°C ± 1°C and humidity maintained at 50% ± 10%. The lights were turned on/off at 8:00 a.m./PM every day. The rats were randomly divided into 6 groups with 8 in each group. The three normal metabolism groups were low-dose, medium-dose, and high-dose crocin I groups. After model establishment, the rats were divided into three groups, namely, low-dose, medium-dose, and high-dose crocin I groups. Similarly, the low, middle, and high dose of the Crocin I group was intragastrically infused with 2.25, 4.5, and 9 mg kg^−1^, respectively.

#### 2.2.2 Plasma sample collection

After the model was established, the rats were gavaged with different doses of crocin I low, medium, and high solutions. 0.5 mL blood was collected from the inner canthus at 0 min, 10 min, 20 min, 30 min, 40 min, 50 min, 1, 2, 3, 4, 6, 8, 10, 12, and 24 h. Centrifuge for 10 min immediately using a refrigerated centrifuge (5,000 rpm/min).

#### 2.2.3 Sample processing and analysis

Take 225 μL of plasma sample at each period and add an equal volume of 225 μL of methanol. Then, 50 μL of the internal standard solution was added, vortexed for 3 min, 12,000 rpm, 4°C, centrifuged for 10 min, and the supernatant was taken for later use. For the preparation of standard solution and internal standard solution, please refer to SI 2.2. The samples were analyzed by high performance liquid chromatography. The relevant analysis conditions are shown in Supplementary Material S2.3. The mobile phase ratio was shown in Supplementary Table S3.

#### 2.2.4 Methodology

The procedures for precision, stability, limit of quantitation, and sample recovery are described in the Supplementary Material S2.4 Methodological Investigations section.

#### 2.2.5 Data processing and statistical analysis

The non-compartmental model of rat plasma at different time points was fitted using DAS 2.0 to obtain the main pharmacokinetic parameters. SPSS 27.0 software was used for statistical analysis of the experimental data, and Origin 2022 software was used for drawing. P < 0.05 was considered statistically significant.

### 2.3 Surface enhanced Raman detection analysis

#### 2.3.1 Procedure for preparing AgNPs

AgNPs were prepared by reducing silver nitrate with sodium citrate (Hou et al., 2017). Accurately measure 380 mL of double distilled water and add it to a 1,000 mL conical flask. Weigh and add 0.36 g of polyvinyl pyrrolidone (PVP) into a conical flask and dissolve it by ultrasonication for 20 min. Accurately weigh 0.3 g of silver nitrate and 0.2 g of sodium citrate, add them to 10 mL centrifuge tubes, and make to 10 mL to prepare silver nitrate solution and sodium citrate solution. Add silver nitrate solution to the Erlenmeyer flask and stir with a magnetic stirrer at 500 rpm for 10 min until the solution is fully dispersed. Finally, the reducing agent sodium citrate solution was added and the reaction was continued for 1 h. AgNPs were obtained by centrifugation washing three times (12,000 rpm) with anhydrous ethanol and double distilled water, each time for 10 min. Freeze-dry and store in a refrigerator at 4°C for later use.

#### 2.3.2 Sample processing and analysis

The treatment of plasma samples is described in Procedure 2.2.3. 50 uL of treated plasma was added into silver nanoparticle solution with an equal volume concentration of 10 mg/mL 50 uL mixed solution was placed on a round quartz sheet with a diameter of 1 cm, 50uL of the mixed solution was placed on a circular quartz plate with a diameter of 1 cm, and the Raman spectrum was detected and recorded. Detection conditions: scanning time 3 s, laser power: 80 mW. Spectra are acquired by Optosky Raman microscopy software.

#### 2.3.3 Methodology

Same procedure for methodological review 2.4.

## 3 Results and discussion

### 3.1 Preventive intervention of crocin I on anxiety-like depression in rats

#### 3.1.1 Crocin I improves weight loss in rats

**FIGURE 1:**
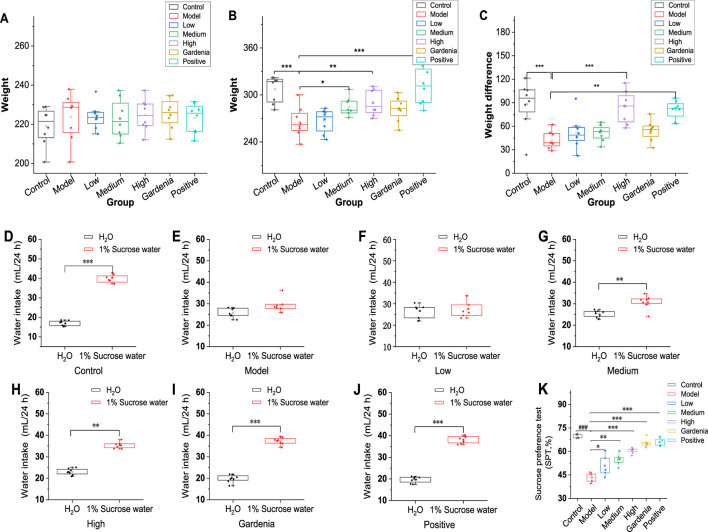
Analysis of body weight differences and sugar water preference rates of rats in each group. **(A)** Initial body weight; **(B)** Body weight after modeling; **(C)** Body weight change; Note: **(D)** The control group; **(E)** Model group; **(F)** Crocin I low dose group; **(G)** Crocin I medium-dose group; **(H)** Crocin I high dose group; **(I)** *Gardenia jasminoides* Ellis extract group; **(J)** The positive drug group; **(K)** Analysis chart of sugar water preference rate of rats in each group Compared with rats in the model group. *P < 0.05,**P < 0.01,***P *< 0.001*).

The rats in each group were weighed and recorded once a week during the whole experiment, recorded as WO, W1, W2, W3, W4, W5. The weekly weight gain and decrease of each rat were calculated. As can be seen from Figures 1A–C, the changes in body weight of rats before and after 1 week of adaptation were negative. The reasons may be related to environmental changes, changes in drinking water quality, and peer changes. The body weight of rats increased significantly in the second, third, and fourth weeks. The change in body weight in each group was negative in the fifth week, which was mainly caused by the Morris water maze test. Compared with the changes in body weight of rats in the same period, the changes in body weight in the low-dose group and model group were lower than those in the middle and high-dose groups, gardenia extract group, and positive drug group.

#### 3.1.2 Crocin I improves anhedonia in rats

As can be seen from Figure 1D, the rats in the control group showed a strong interest in sucrose solution, and the results were consistent with the experimental hypothesis. The rats in the model group showed no significant differences in water and sucrose solutions (Figure 1E). As shown in Figures 1F–H, the rats were dose-dependent to different doses of Crocin I. The rats in the gardenia extract group (Figure 1I) showed a higher demand for sugar water, and the sugar water preference index was 65.3%. Similarly, the rats in the positive drug group (Figure 1J) also showed a sugar preference, and the sugar preference index was 66.0%, while the sugar water preference index of the model group was significantly lower than that of other groups, and the result was 43.3%, with a significant difference (Figure 1K). The results showed that the experimental rat model was successful, and showed different degrees of sugar preference under the intervention of low, medium, high, gardenia extract and positive drug paroxetine. The preference for sugar water has been improved in varying degrees, and it also shows the intervention effect of these drugs on the behavior of rats with Anxiety-like depression.

#### 3.1.3 Crocin I improved long-term neurological function

**FIGURE 2:**
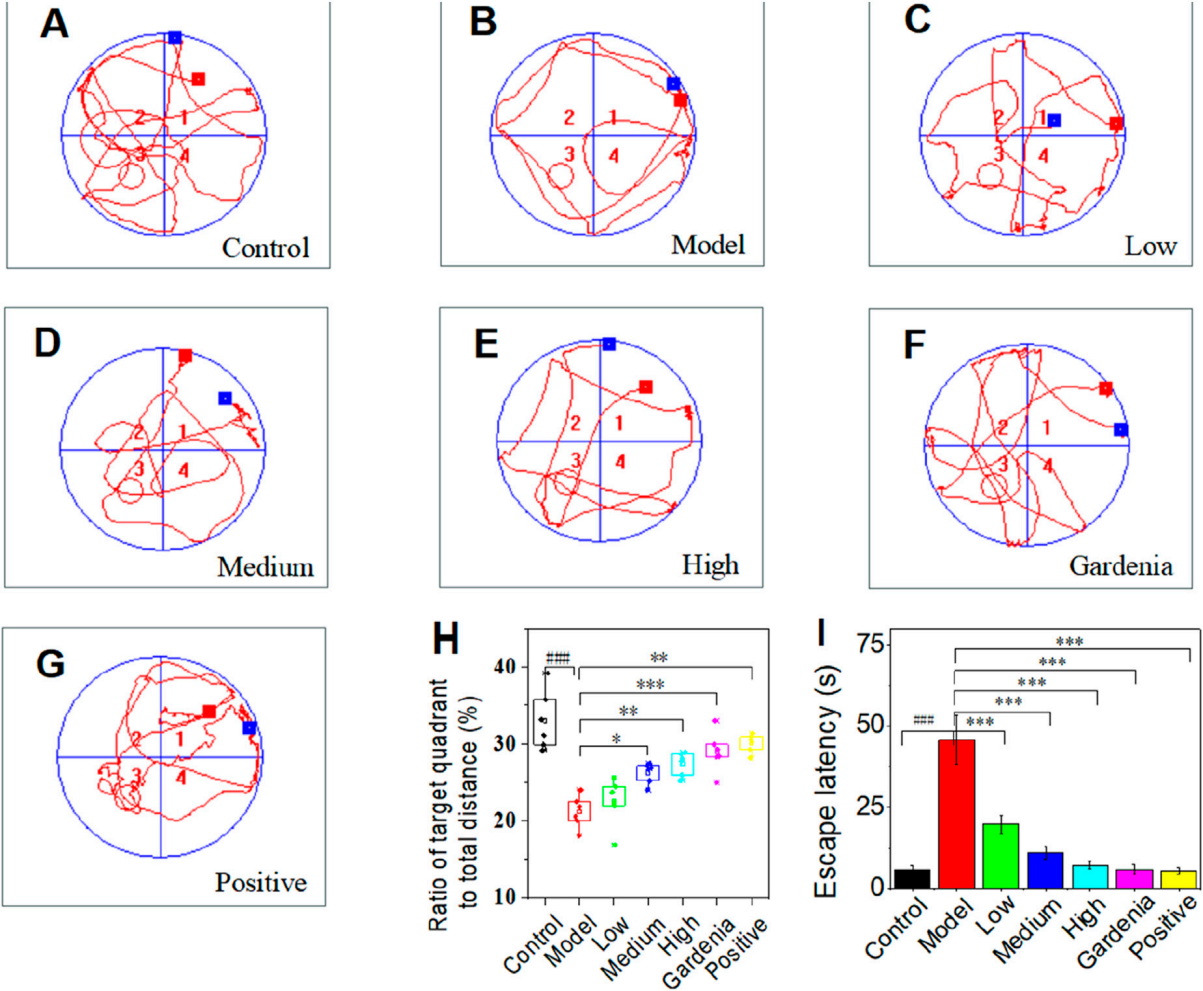
Space exploration trajectory map of the water maze in each group of rats **(A)** Control group; **(B)** Model group; **(C)** Crocin I low dose group; **(D)** Crocin I medium-dose group; **(E)** Crocin I high dose group; **(F)** Gardenia jasminoides Ellis extract group; **(G)** Positive drug group; **(H)** The percentage of target quadrant distance to total distance; **(I)** Escape incubation period. Note: ^#^P < 0.05, ^##^P < 0.01, ^###^P < 0.001, vs. Control; *P < 0.05,**P < 0.01,***P < 0.001, vs. Model.).

The water maze test is an important way to evaluate the learning, cognition, and memory ability of rats with anxiety-like depression. Following subcutaneous injection of corticosterone in SD male rats, we then performed the Morris water maze test, which is closely associated with a range of depressive-like phenotypes to assess anxiety-like phenotypes. As shown in Figures 2A–C, with the increase in training times and time, the time of rats searching for underwater hidden target platforms gradually shortened, and the escape latency decreased significantly. It presented the change law of edge type-trend type-linear type. Figures 2A–G shows that after training, the underwater concealment platform is removed and the rats cross the target quadrant. It could be observed that, compared to the other groups, the model group exhibited fewer crossings of the target quadrant and shorter displacements.

The rats in the control group showed a trend search, while most of the rats in the model group showed a marginal search (Figures 2A, B). After the intervention of Crocin I and paroxetine, there was a tendency to look for it (Figures 2A–C). In addition, the spatial cognitive ability of rats in each group can be accurately judged by the ratio of the target quadrant distance to the total distance (Figure 2H) and the escape latency (Figure 2I) in the water maze experiment. Therefore, we draw the experimental data of rats in each group. As can be seen from the chart, the data in Figures 2H, I confirm each other and have a high degree credibility. The ratio of the target quadrant to total distance increased (Figure 2H) and the escape latency decreased (Figure 2I) in low, middle, and high dose groups. It is confirmed that Crocin Ⅰ can improve the spatial memory ability of rats in a dose-dependent manner. Compared with the model group and the control group, there were significant differences in other groups.

#### 3.1.4 Crocin I alleviates hippocampal morphological changes

**FIGURE 3:**
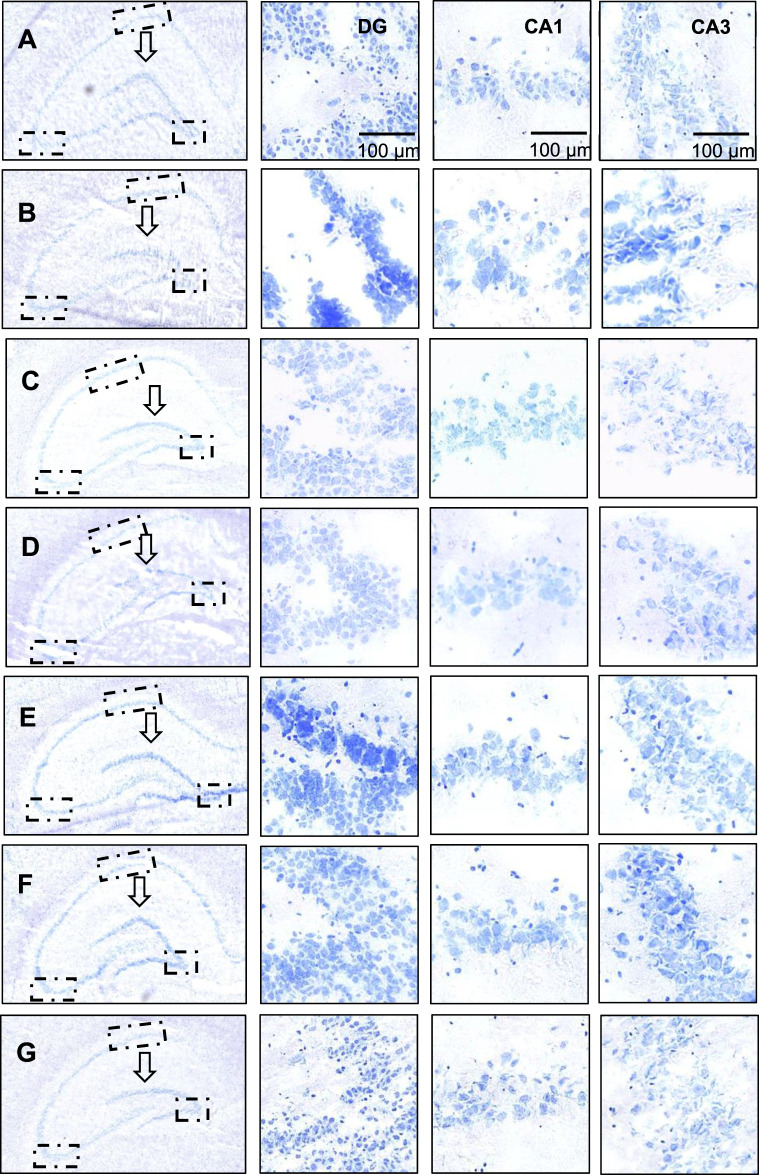
Nissl staining analysis of hippocampal neurons in rats. **(A)** The control group; **(B)** Model group; **(C)** Low dose group; **(D)** Medium dose group; **(E)** High dose group; **(F)** Gardenia jasminoides Ellis extract group; **(G)** Positive drug group).

As shown in Figures 3A–G (black arrows), the dentate gyrus of the control group has clear and sharp edges, while the dentate gyrus of the model group (Figure 3F) has blunted edges, smoother and gentler. As the dose of crocin I increased (low, medium, and high), the dentate gyrus angle showed a sharper trend. The low-dose group showed no significant change, while the middle- and high-dose groups showed significant changes. The shape gradually approached that of the control group. In contrast, the dentate gyrus morphology of the gardenia extract group was almost the same as that of the control group, which strongly proves that both crocin I and gardenia extract can effectively prevent corticosterone-induced morphological changes in the dentate gyrus of the hippocampus. And the effect is remarkable. However, similar improvements were not observed in the positive drug group. These results indicated that, unlike the positive drug group, both the crocin I and gardenia extract groups had a significant improvement effect on abnormal hippocampal morphology. This is also the first time that the preventive intervention effect of crocin I on rat hippocampal morphology has been observed, reflecting the advantages of crocin I compared with positive drugs.

#### 3.1.5 Crocin I alleviates hippocampal neuronal damage

##### 3.1.5.1 Dentate gyrus (DG) of the hippocampus

As shown in Figure 3, the nucleoli of the neurons in the dentate gyrus of the hippocampus of the rats in the control group were obvious, with clear boundaries, abundant Nissl bodies, and intact neurons. The hippocampal neurons of the rats in the model group (Figure 3B) were damaged, showing blurred boundaries, nuclear fragmentation, nuclear lysis, and a decreased number of Nissl bodies. This indicated that the model was successfully established. The neuronal cell damage in the dentate gyrus region was significantly improved after the administration of crocin I, gardenia extract, and the positive control drug. The positive drug group could almost restore the neuronal cell damage to the level of the control group. In terms of the number of neuronal cells, it was observed that the number of Nissl bodies increased significantly in the high-dose crocin I and gardenia extract groups. These findings strongly prove that the crocin I high-dose group, gardenia extract group, and positive drug group can effectively intervene in corticosterone-induced hippocampal neuron damage, thereby alleviating anxiety-like depression symptoms in rats.

##### 3.1.5.2 Hippocampus Cornu Ammonis 1 (CA1)

Compared with the DG area, neuronal cell damage in the hippocampal CA1 area was less significant. In the CA1 region images shown in Figures 3A–G, it can be seen that, except for the control group (Figure A) which maintained the integrity of neuronal cells, the other groups suffered varying degrees of damage. Especially in the model group (Figure 3B), neuronal cells were severely damaged, cell morphology was highly changed, boundaries were blurred, and typical cell apoptosis characteristics such as nuclear condensation, nuclear fragmentation, and nuclear dissolution appeared. However, the damaged neuronal cells showed varying degrees of improvement, and the number of Nissl bodies also significantly increased after the administration of high doses of crocin I, gardenia extract, and the positive control drug. Nonetheless, the improved state of the neuronal cells still did not fully restore to the healthy levels observed in the control group.

##### 3.1.5.3 Hippocampus cornu ammonis 3 (CA3)

Compared with the hippocampal dentate gyrus and CA1 area, corticosterone causes more direct and significant damage to the hippocampal CA3 area, and the damage to neurons and cells is intensified. After administration of crocin I, the number of Nissl bodies increased in a dependent manner in the low, medium, and high dose groups. The number of Nissl bodies in the Gardenia extract group was significantly restored and was better than that in the high-dose crocin I group. Although the damage status of neuronal cells in the positive drug group was significantly improved, there was no significant increase in the number of Nissl bodies. These results suggest that crocin I, gardenia extract, and positive drugs can effectively alleviate the damage of hippocampal neurons in anxious and depressed rats induced by corticosterone. Among them, crocin I and gardenia extract can also promote the increase in the number of hippocampal neurons in the CA3 area of rats, thereby exerting its therapeutic effect on anti-anxiety-like depression.

Taking together, the degree of damage caused by corticosterone to various regions of the rat hippocampus (DG area, CA1 area to CA3 area) showed an increasing trend. After administration of crocin I, gardenia extract, and positive drugs, all the above-mentioned damaged areas showed significant improvement effects. Specifically, crocin I and gardenia extract performed particularly well in repairing hippocampal morphological damage. In contrast, the positive drug group showed no obvious morphological recovery changes. In terms of the repair of neuronal cell damage, the high-dose crocin I group, the gardenia extract group, and the positive drug group all achieved significant results, among which the degree of improvement in the positive drug group was closest to the level of the normal control group. Furthermore, in terms of promoting the recovery of the number of neurons, both the high-dose crocin I group and the gardenia extract group were able to effectively increase the number of neurons in various areas of the hippocampus, demonstrating its positive role in the treatment of anxiety and depression-related neuronal damage.

#### 3.1.6 Crocin I improves the survival rate of rat cortical neurons

The rats were randomly divided into seven groups, about the administration of the reference positive medicine and different doses and types of preventive intervention drugs, namely, Crocin 2.25 mg kg^−1^, Crocin 4.5 mg kg^−1^, Crocin 9 mg kg^−1^ and Gardenia jasminoides 0.9 g kg^−1^ (n = 56, in total).

**FIGURE 4:**
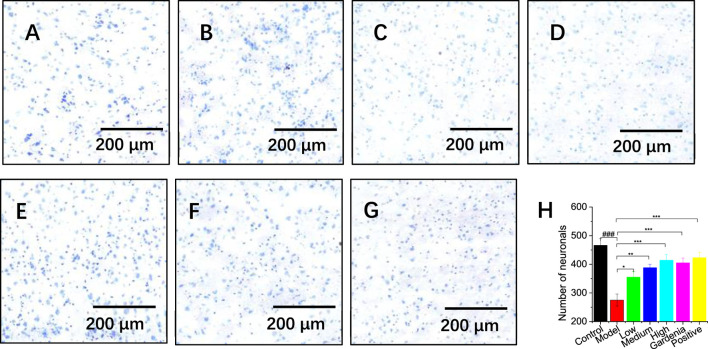
Effects of different groups of rats on the prefrontal cortex **(A)** The control group; **(B)** Model group; **(C)** Low dose group; **(D)** Medium dose group; **(E)** High dose group; **(F)** *Gardenia jasminoides* Ellis extract group; **(G)** Positive drug group; **(H)** Analysis of hippocampal neuron numbers; Note: ^#^P < 0.05, ^##^P < 0.01, ^###^P < 0.001, vs. Control; *P < 0.05,**P < 0.01,***P < 0.001, vs. Model.).

The prefrontal cortex plays an important role in participating in memory cognition and regulating emotions, and movement. The experiment performed Nissl staining on the prefrontal cortex of rats. The results are shown in Figures 4A–H. The pyramidal cells in the control group were clearly layered, orderly in structure, regular in shape, with clear nuclei and abundant Nissl bodies. Blue plaque-like Nissl bodies were visible. The neuronal cells in the model group had irregular shapes and blurred boundaries, some Nissl’s cells were significantly dissolved and disappeared, and the pyramidal cells showed nuclear pyknosis and nuclear dissolution.

In the high-dose crocin I group, gardenia extract group, and positive drug group, the color of the Nissl bodies significantly darkened and their density increased (Figures 4E–G). This suggested enhanced neuronal cell expression and significant repair of damage. ImageJ software was used to conduct an in-depth analysis of hippocampal neuron cells (as shown in Figure 4H). The results showed that the number of cortical neurons in the model group was significantly reduced compared with that in the control group (***P < 0.001), confirming that the model was successfully established. Further observation found that as the dosage of crocin I increased (low, medium, and high doses), the number of Nissl bodies also increased, which fully demonstrated the protective effect of crocin I to cortical neuron cells. At the same time, an increase in the number of Nissl bodies was also observed after treatment with Gardenia extract and positive drugs. This shows that the Gardenia extract group and the positive drug group can effectively prevent the massive apoptosis of cortical neurons and play a neuronal protective role.

#### 3.1.7 Crocin I improves the regulation of monoamine neurotransmitter levels

**FIGURE 5:**
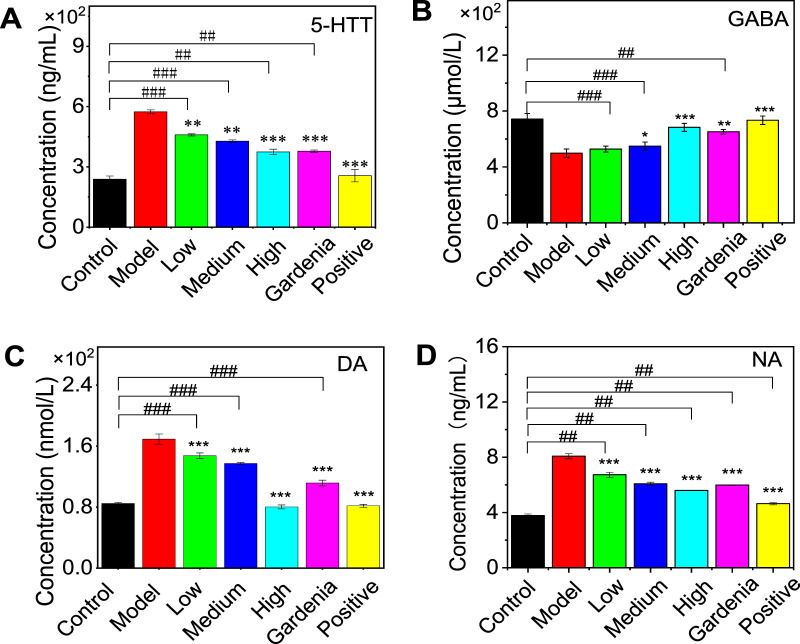
Effects of Crocin Ⅰ and *Gardenia jasminoides* Ellis extract on the expression of 5-HTT **(A)**, GABA **(B)**, DA **(C)**, and NA **(D)** in the brain of rats with anxiety depression. (1. Control group 2. Model group 3. Low dose group 4. Medium dose group 5. High dose group 6. Gardenia extract group 7. Positive drug group Note:, ^#^P < 0.05, ^##^P < 0.01, ^###^P < 0.001, vs. Control; *P < 0.05,**P < 0.01,***P < 0.001, vs. Model).

The expression levels of 5-HTT, GABA, DA, and NA in the brain tissue of anxious and depressed rats were determined by ELISA. As shown in Figure 5, compared with the control group, the content of 5-HTT in the model group increased significantly; there was no significant difference in the positive drug group. This indicates that the positive drug group can restore the 5-HTT content in the rat brain to normal. Compared with the model group, the content of 5-HTT in the crocin I low-, medium-, and high-dose groups, gardenia extract group, and positive drug group were significantly reduced (P < 0.001), indicating that gardenia extract and crocin Anthocyanin I can improve the overexpression of 5-HTT in the brain tissue of depressed rats to varying degrees, inhibit the reuptake process mediated by 5-HTT, and thereby increase the 5-HT content in the synaptic cleft.

As for the changes in GABA content in the brain of rats in the model group, it can be seen from the figure that the low, medium, and high dose groups of crocin I showed a dependent decrease. There was no significant difference in the low-dose group, indicating that it was difficult for the low-dose group to significantly improve the GABA content in the rat brain. The medium and high-dose crocin I group, gardenia extract group, and positive drug group could significantly reduce the GABA content in the brains of rats. Compared with the control group, there was no significant difference in the improvement of GABA content by gardenia extract, suggesting that gardenia extract can restore the GABA content in depressed rats to normal levels.

Compared with the model group, the dopamine (DA) and norepinephrine (NA) contents in the rat brain were significantly increased in other groups (P < 0.001). Compared with the control group, there was no significant difference in brain DA between the crocin I high-dose group and the positive drug group, indicating that these two groups could restore the rat brain to normal levels.

The above results show that after drug intervention, the levels of 5-HTT, GABA, DA, and NA in the brains of anxious and depressed rats changed to varying degrees (as shown in Table 1). Compared with the model group, except for the low dose of crocin I, which had no significant difference in GABA content, all other groups could significantly improve the levels of 5-HTT, GABA, DA, and NA in the rat brain. Among them, the positive drug group could restore 5-HTT in the rat brain, GABA in the gardenia extract group, and DA in the high-dose crocin I group, and the gardenia extract group could restore to normal levels.

**TABLE 1:** Expression levels of 5-HTT, GABA, DA, and NA in brain tissue of rats in each group.

Group	5-HTT (ng/mL)	GABA (µmol/L)	DA (nmol/L)	NA (ng/mL)
Control	238.58 ± 16.09	7.43 ± 0.39	84.66 ± 1.749	3.78 ± 0.10
Model	573.69 ± 9. 80	4.99 ± 0.298	169.17 ± 6.64	8.08 ± 0.19
Low	374.76 ± 13.24	5.28 ± 0.21	147.33 ± 3.72	5.99 ± 0.02
Medium	459.21 ± 5.21	5.49 ± 0.29	137.22 ± 1.31	6.73 ± 0.18
High	427.64 ± 7.00	6.83 ± 0.29	80.22 ± 2.64	5.60 ± 0.02
Gardenia extract	378.07 ± 5.96	6.52 ± 0.17	111.54 ± 3.73	6.08 ± 0.12
Positive	256.14 ± 30.37	7.34 ± 0.30	81.70 ± 1.94	4.64 ± 0.09

### 3.2 Pharmacokinetic analysis of crocin I

**FIGURE 6:**
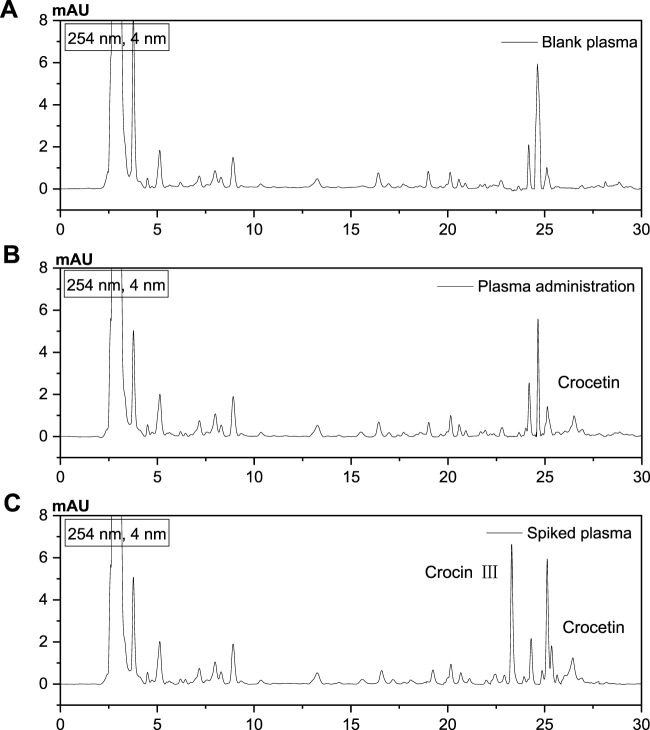
Analysis of metabolites by HPLC. **(A)** Blank plasma, **(B)** After administration of Crocin I, plasma, **(C)** Standard plasma).

To understand the metabolism and absorption of crocin I in the body, the *in vivo* pharmacokinetics of the active ingredient crocin I was studied. The results showed that no crocin I was detected in the rat plasma. It is speculated that crocin I cannot pass through the intestinal barrier and enter the blood of rats. This discovery does provide a new perspective for research in related fields. The reason may be that crocin I is a highly unsaturated carotenoid with poor stability and is sensitive to high temperature, light and low pH value. Its oil-water partition coefficient (logP) is −1.03, which is far lower than the range of logP value (2–3) which is generally considered to be easily absorbed in the intestine. Therefore, crocin I is not easily absorbed in the intestine. The results show that the apparent permeability coefficient Papp of crocin I is low. It is further confirmed that it cannot penetrate the intestinal barrier. Different from crocin I, crocetin lacks four sugar groups in its skeleton structure. The polarity of the molecule is greatly reduced, and the fat solubility is increased. Therefore, it can permeate Caco-2 cells at a faster rate. And that apparent permeability coefficient (Papp) value is high. It shows that it can penetrate the intestinal barrier and be well absorbed in the intestine. Because crocin I can't pass through the intestinal barrier, its bioavailability after oral administration is relatively low. Although crocin I itself is not easily absorbed, it will be hydrolyzed into crocetin in the gastrointestinal tract and then absorbed into the blood circulation. It is suggested that we can consider developing drugs with crocetin as the main active ingredient. Alternatively, the bioavailability of crocin I can be improved by changing the dosage form of the drug (such as injection) or using a new drug delivery system. In addition, the study also found that hypoxic environment has a certain promoting effect on the absorption of crocin Ⅰ. This may provide new ideas for drug development under specific conditions (such as plateau environments). Subsequently, its metabolites were further analyzed and tested, and its metabolic component was found to be crocetin. It is speculated that its metabolite crocetin can pass through the intestinal barrier and enter the blood system. As shown in Figure 6.

#### 3.2.1 Analysis of crocin I metabolites

Its metabolic mechanism was studied using *in vitro* acid hydrolysis experiments, and its metabolites were detected by LC-MS. Several related products were found. In the negative ion mode, the quasi-molecular ion peak of the first metabolite is m/z 813.81. The secondary mass spectrum fragment ion peaks are m/z 650.95 [M-H-C_6_H_10_O_5_], and 327.10 [M-H-2C_6_H_10_O_5_]. By comparing with literature data (Zhuang et al., 2023), compound (P1) was identified as crocin II.

The second metabolite (P2) has a quasi-molecular ion peak of m/z 651.16 and a secondary mass spectrum fragment of m/z 327.10. They were identified as crocin III (P2) and crocin IV (P3), respectively. Similarly, the third metabolite (P4) has a quasi-molecular ion peak of m/z 327.15 and a fragment ion peak of m/z 283.46 [M-H-CO_2_]. P4 was identified as crocetin. The primary and secondary mass spectra of metabolites are shown in Supplementary Figures S2–S6, respectively. The metabolite list analysis is shown in Table 2.

**TABLE 2:** List of Crocin I metabolites.

No.	t_R_/min	MW	Precursor ion (m/z)	Compound
A1	6.384	814	813 [M-H]^-^	Crocin II
A2	5.102	701.52	702.75 [M + H]^+^	Unknown
A3	5.071	652	651 [M-H]^-^	Crocin III
A4	8.093	652	651 [M-H]^-^	Crocin IV
A5	4.024	588.56	589.79 [M + H]^+^	C_28_H_42_O_14_
A6	2.727	472	473 [M + H]^+^	C_26_H_32_O_8_
A7	1.366	416.63	417 [M + H]^+^	C30H40O
A8	10.082	327	350.78 [M + Na]^+^	Crocetin

#### 3.2.2 Analysis of metabolic pathway of crocin Ⅰ

**FIGURE 7:**
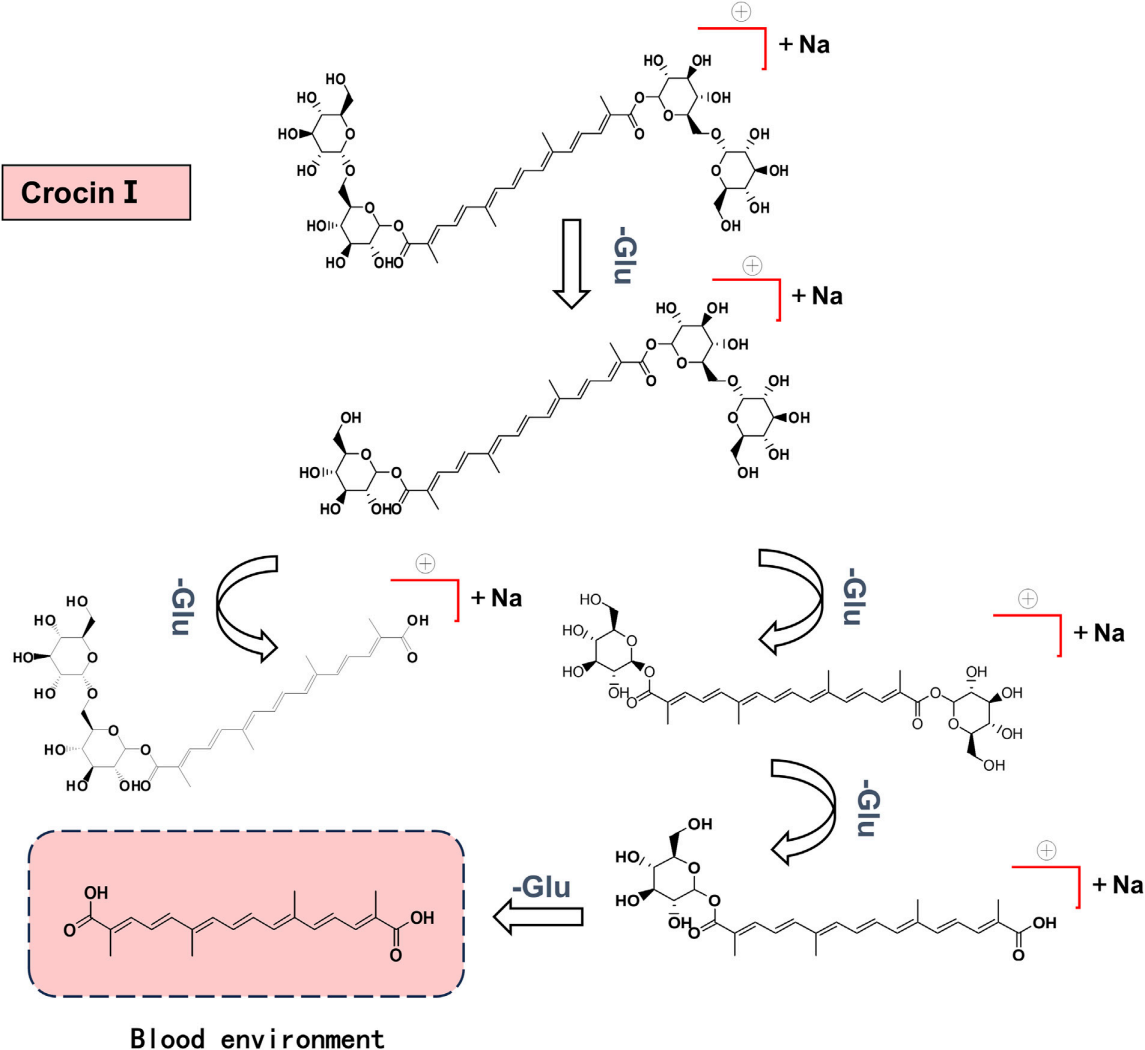
Crocin Ⅰ cleavage pathway in rats.

Metabolites were analyzed based on the parent ion and daughter ion fragment information. It was found that the cleavage of crocin I was through the cleavage of glycosidic bonds, generating different metabolites in turn. It is ultimately metabolized into crocetin, which contains only four isopentenyl structures. The speculated *in vivo* cleavage pathway of crocin I is shown in Figure 7.

#### 3.2.3 Methodology

##### 3.2.3.1 Specificity analysis of metabolite crocetin

In this experiment, crocetin, a metabolite of crocin I, was selected as the index component for *in vivo* pharmacokinetic testing. Crocin III, which has the same structural core component, was used as the internal standard for content analysis. The standard curves of crocetin and crocin III are shown in Supplementary Figure S7. By comparing blank plasma, drug-treated plasma, and spiked plasma, it was found that this method has good specificity. The internal standard crocin III does not affect the analysis and determination of crocetin. The relevant spectrum is shown in Figure 6.

##### 3.2.3.2 Precision, stability, LOQ, and sample recovery rate

The results of the methodological investigation showed that the RSD (1%–8%) of low, medium, and high doses of crocetin within 1 week were all less than 15%, with good precision. The accuracy (94%–101%) was between 85%–115%, which was a good accuracy. Stability results showed that the accuracy of plasma samples was (97%–100%) and stable within 1 week. The Limit of quantification (LOQ) was 31.25 ng/mL. The sample recovery rate is between 95% and 105%, which meets the analysis requirements of samples in plasma. The results are shown in Tables 3–5, respectively.

**TABLE 3:** Experimental results of precision and accuracy of crocetin (n = 6).

Concentration	Measured concentration	Precision	Accuracy
(ng/mL)	(ng/mL)	(RSD, %)	(RE, %)
292.60	289.16 ± 45.8	2.90	98.40
527.32	517.86 ± 36.5	7.37	94.03
837.96	844.32 ± 64.3	1.23	100.26

**TABLE 4:** The experimental results of the stability of crocetin (n = 6).

Study dose	Concentration	Measured concentration	Accuracy
	(ng/mL)	(ng/mL)	(RE, %)
Low dose	292.60	285.47 ± 31.7	97.76
Medium dose	527.32	512.28 ± 47.3	97.14
High dose	837.96	834.94 ± 43.4	99.64

**TABLE 5:** The experimental results of the recovery rate of crocetin (n = 6).

Study dose	Concentration	Recovery rate	
	(ng/mL)	Mean ± SD	(RSD, %)
Low dose	292.59	99.42	3.66
Medium dose	527.32	99.10	3.31
High dose	837.96	101.6	0.87

#### 3.2.4 Determination of metabolite crocetin content

**FIGURE 8:**
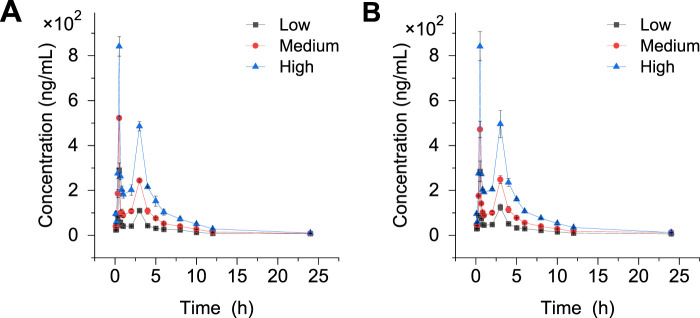
Drug-time curves before and after modeling. **(A)** Normal Metabolome **(B)** Model metabolome.

The crocetin content was determined at 0 min, 10 min, 20 min, 30 min, 40 min, 50 min, 1, 2, 3, 4, 6, 8, 10, 12, and 24 h after administration. The standard curves of crocetin and internal standard crocin III are shown in Supplementary Figure S2. Draw the drug-time curve (Figure 8). It can be seen that crocetin metabolite crocetin I reaches peak value at different periods in rats. This suggests that the drug may have inconsistent absorption sites in the stomach, enterohepatic circulation, or inconsistency between gastric juice pH and gastric emptying rate.

The DAS2.0 fitting model was used and the statistical distance method was used to calculate the parameters. The pharmacokinetic parameters of Crocin I after gavage administration in rats are shown in Table 6. The results demonstrated that following gavage administration of low, medium, and high doses of Crocin I, the half-life of Crocin was 3.75 ± 0.06 h. The time to peak concentration was 0.5 h. The area under the plasma concentration-time curve varied between 521.73 and 2,243.98 mg·L/h. The mean residence time ranged from 5.09 to 6.21 h. The results showed that there was a good linear dose-response relationship for crocetin in the low, medium, and high dose groups. In terms of clearance rate, both the low- and medium-dose groups showed lower clearance rates than the normal metabolism low- and medium-dose groups, indicating that the modeling may have caused a certain degree of damage to the liver and kidneys of SD rats, thereby slowing down the metabolic process of crocin I. In the high-dose group, the clearance rates of crocin Ⅰ in the normal group and after modeling were both 0.009 h, indicating that high-dose crocin Ⅰ can significantly improve the impact of modeling on the metabolic clearance rate of rats and restore it to a normal level.

**TABLE 6:** Pharmacokinetic parameters of Crocin Ⅰ after intragastric administration in rats.

Parameter	Unit	Normal metabolism			Metabolism after modeling		
		Low dose	Medium dose	High dose	Low dose	Medium dose	High dose
AUC_(0-t)_	mg·L/h	521.728	1,091.244	2,102.38	528.931	1,139.487	2,224.980
AUC_(0-∞)_	mg·L/h	527.165	1,126.271	2,183.281	532.272	1,213.048	2,243.978
MRT_(0-t)_	h	5.247	5.120	5.093	5.256	5.46	5.314
MRT_(0-∞)_	h	5.848	6.205	6.093	6.128	6.17	6.112
t_1/2_	h	4.124	3.605	3.778	3.684	3.516	3.786
T_max_	h	0.5	0.5	0.5	0.5	0.5	0.5
CL	L/h/kg	0.009	0.013	0.009	0.007	0.012	0.009
C_max_	mg/L	290.880	522.588	837.960	300.985	471.548	841.391

### 3.3 Surface enhanced Raman (SERS) analysis

Based on an in-depth study of crocetin metabolism *in vivo*, the experiment conceived a cutting-edge hypothesis: By integrating surface-enhanced Raman spectroscopy, can we conduct a deeper analysis of metabolites (crocetin) in plasma, thereby achieving high-precision detection and identification of target substances?

#### 3.3.1 Characterization of nanosilver substrate materials

**FIGURE 9:**
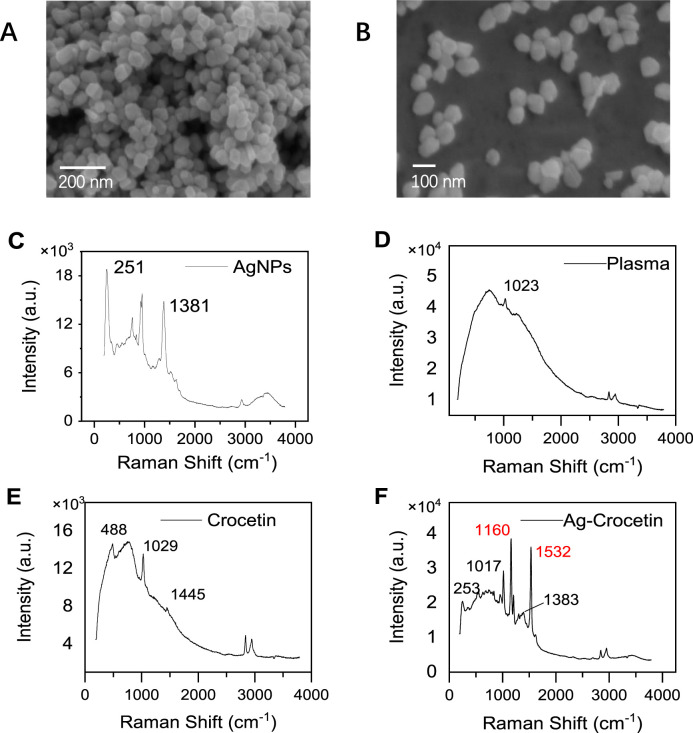
Scanning electron microscopy (SEM) and Raman spectroscopy. **(A, B)** AgNPs (SEM) **(C)** AgNPs (SERS) **(D)** Plasma **(E)** Crocetin **(F)** Ag- Crocetin).

According to literature records, silver nanoparticles with a particle size of 70 nm will exhibit a strong surface enhancement effect (Stamplecoskie et al., 2011). In this study, silver nanoparticles with an average particle size of 81.05 nm were synthesized, and the scanning electron microscopy results are shown in Figures 9A, B. The Raman spectrum of the silver nanoparticles was collected, as shown in Figure 9C.

#### 3.3.2 Analysis of plasma samples

The silver nanoparticles synthesized by this method were further used to detect crocetin in plasma. Figures 9D, E depict the Raman spectra signals of plasma and crocetin in the absence of silver nanoparticle enhancement, respectively. We observed that the peak shapes of both signals were lower and the characteristic peak intensities were weaker. In particular, in Figure 9E, the characteristic peaks of crocetin at 1,162 cm^−1^ and 1,532 cm^−1^ were almost invisible, and only the absorption peaks of methanol at 1,029 cm^−1^ and 1,045 cm^−1^ were visible. This indicates that the Raman scattering of crocetin is extremely weak at this concentration. After enhancement by silver nanoparticles, as shown in Figure 9F, the peak shape is improved and the signal intensity is enhanced. In particular, the signal peaks at 1,162 cm^−1^ and 1,532 cm^−1^ are attributed to the C-C stretching vibration and C=C stretching vibration, respectively. It was finally identified as the characteristic peak of saffron (Kyriakoudi et al., 2024).

#### 3.3.3 Stability, detection limit, enhancement factor calculation

**FIGURE 10:**
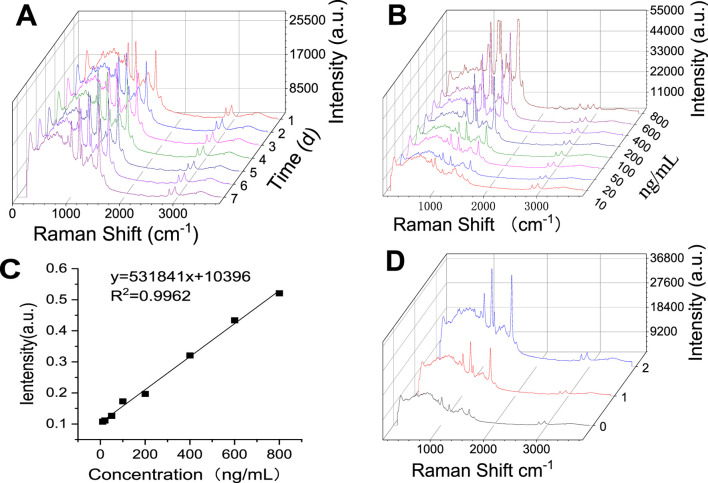
Investigation of the Methodology of Surface Enhanced Raman Detection of Crocetin. **(A)** Stability **(B)** Detection limit **(C)** Standard curve of crocetin **(D)** Raman spectra of low, medium, and high concentrations of crocetin).

The stability of this SERS method was tested by examining the peak profile and intensity of crocetin over a week using a reference standard. As shown in Figure 10A, we observed that the representative spectra had almost no change in intensity at 1,532 cm^−1^, and the contours overlapped with each other. This indicates that the method is stable for at least 1 week, which is sufficient for testing because the protocol can be completed in just minutes.

The characteristic peak of crocetin at 1,532 cm^−1^ was selected for detection limit calculation. The detection limit was defined as the concentration at which the signal-to-noise ratio was 3:1. Preparation of a series of crocetin solutions: 10 ng/mL, 20 ng/mL, 50 ng/mL, 100 ng/mL, 200 ng/mL, 400 ng/mL, 600 ng/mL, and 800 ng/mL. Each concentration was sampled 6 times to obtain the average intensity of the characteristic peak 1,532 cm^−1^, as shown in Figure 10B. This peak has a good linear relationship in the range of 10 ng/mL-800 ng/mL, as shown in Figure 10C. When the signal-to-noise ratio was 3:1 and the concentration was 10 ng/mL, the sensitivity was good, confirming the possibility of being used to detect trace amounts of crocetin.

The enhancement of crocetin was investigated by a published method (Lee et al., 2021; Zhao et al., 2024; Zhou et al., 2024). Screening was performed based on the resolution, and the peak at 1,532 cm^−1^ was determined to be the main absorption signal for investigation. Taking inspiration from the literature, we calculated the enhancement factor by Equation 1 (Bell et al., 2020).

Enhancement factor calculation formula:

$$EF=\frac{I_{SERS}\times N_{NC}}{I_{NC}\times N_{SERS}}$$ (1)

Where N_NC_ represents the effective number of laser-irradiated crocetin molecules in the solution. N_SERS_ represents the number of molecules enhanced on SERS substrates (AgNPs). Likewise, INC and ISERS indicate the intensities of molecules in the normal solution and after SERS enhancement, respectively.

The calculation analysis was performed using the monolayer distribution of crocetin at a concentration of 200 ng/mL, which did not reach the saturation concentration, on the silver nanoparticles. Fifty microliters of 200 ng/mL crocetin was added onto a microscope slide. The diameter of the droplet was measured to be 0.7 cm, and the droplet area was calculated to be 0.38 cm^2^. Calculated based on the spot diameter of 1.622 *μ*m, the laser spot area is 2.066 μm^2^. The effective number of molecules in the laser and solution are 569 and 6.22 × 10^13^, respectively. The calculated enhancement factor is 4.49 × 10^11^.

#### 3.3.4 Quantitative detection of crocetin concentration by SERS based

The sensing ability of the SERS substrate was demonstrated by exposing the SERS substrate to different concentrations of crocetin (10 ng/mL, 20 ng/mL, 50 ng/mL, 100 ng/mL, 200 ng/mL, 400 ng/mL, 600 ng/mL, and 800 ng/mL). A standard curve was prepared based on the peak intensity of the characteristic peak at 1,532 cm^−1^ and the content was determined, as shown in Figure 10C. Taking the 0.5 h medium-dose plasma as an example (Figure 10D), the crocetin content was measured to be 432.89 ng/mL. The same sample was subjected to liquid phase determination, and the content was measured to be 486.03 ng/mL. Both methods exhibited a small range of fluctuation and fully met the requirements for rapid detection of crocetin acid in plasma.

## 4 Conclusion

In this research, we elaborated on three subtopics, including the pharmacodynamic evaluation of crocin I, *in vivo* pharmacokinetic analysis, and SERS rapid detection. We aim to explore new approaches to the treatment of anxiety-like depression and to develop rapid and efficient testing methods. Through research on the anti-anxiety-like depression effect of crocin I, it was discovered for the first time that crocin Ⅰ and gardenia extract can significantly improve the changes in hippocampal morphology caused by corticosterone. It can effectively improve the damage of nerve cells in the DG, CA1, and CA3 regions of the hippocampus and play a neuroprotective role. At the same time, it exerts a therapeutic effect by down-regulating the reuptake of 5-HTT, DA, and NA neurotransmitters in the brain tissue of anxious and depressed rats and up-regulating the expression level of GABA. Pharmacokinetic analysis revealed that crocin I could not pass through the intestinal barrier into the blood to exert an anti-anxiety-like depressive therapeutic effect.The material basis of its action lies in the metabolite crocetin. In terms of trace detection, AgNPs were prepared and synthesized. A new method for detecting trace amounts of crocetin in plasma was established through Raman spectroscopy characterization and methodological investigation. The results reveal the potential of crocin Ⅰ in neuroprotection. It not only provides a new perspective for the administration of crocin Ⅰ, but also lays a certain theoretical foundation for the treatment of anxiety-like depression.

## Data Availability

The original contributions presented in the study are included in the article/Supplementary Material, further inquiries can be directed to the corresponding author.
