# Correction: Reversal of anxiety-like depression induced by chronic corticosterone by crocin I and surface-enhanced Raman spectroscopy monitoring of plasma metabolites

**DOI:** 10.3389/fphar.2026.1883631

**Published:** 2026-06-10

**Authors:** Dandan Zhang, Zhuodi Wu, Doudou Yang, Guanjie Zhao, Yanru Zhang, Weifeng Mou, Yinku Liang

**Affiliations:** School of Biological Sciences and Engineering, Shaanxi University of Technology, Hanzhong, China

**Keywords:** crocin I, anxiolytic-like depression, pharmacokinetics, surface-enhanced Raman spectroscopy, trace monitoring

The **acknowledgment** was erroneously omitted when the article was published. The correct acknowledgment section should be: “We sincerely thank Professor Xinfeng Zhao from Northwest University for providing valuable experimental platform support during this study.”

The original article has been updated.

