# Supplementary material for: Reversal of anxiety-like depression induced by chronic corticosterone by crocin I and surface-enhanced Raman spectroscopy monitoring of plasma metabolites
Source: Front Pharmacol. 2025 Feb 27;16:1540551. doi: 10.3389/fphar.2025.1540551 (PMC11903455; doi:10.3389/fphar.2025.1540551)
Supplement: Supplementary file 1 [file DataSheet1.doc]

***Supporting information for:***

Reversal of anxiety-like depression induced by chronic corticosterone by crocin I and surface-enhanced Raman spectroscopy monitoring of plasma metabolites

Dandan Zhang, ^#^ Zhuodi Wu,^#^  Doudou Yang, Guanjie Zhao, Yanru Zhang,
Weifeng Mou, Yinku Liang*

School of Biological Sciences and Engineering, Shaanxi University of Technology, Hanzhong 723000, China

*Corresponding author: Yinku Liang

Fax: (+86) 0912125002

Tel: (+86) 13892607820

Email: liangyinku26@163.com

Table S1 Experimental materials and reagents

| Materials and reagents | Manufacturer | Purity |
| --- | --- | --- |
| Paroxetine Hydrochloride | Changzhou Watson Pharmaceuticals Co., Ltd. | 98% |
| DMSO | Tianjin Komiou Chemical Reagent Co., Ltd. | 99% |
| Methanol | Jinan Exxon Chemical Co., Ltd. | 99.5% |
| Fetal bovine serum | Beijing Diyi Biotechnology Co., Ltd. | / |
| Penicillin | Shanghai Zeye Biotechnology Co., Ltd. | 99% |
| Streptomycin | Weiqi Biotechnology Co., Ltd. | 99% |
| PMSF | Yisheng Biotechnology (Shanghai) Co., Ltd. | 98% |
| RIPA | Shanghai Xinfan Biotechnology Co., Ltd. | 99.9% |
| Corticosterone | Hubei Kangbaotai Fine Chemical Co., Ltd. | 99% |
| Crocin I | Chengdu Pusi Biotechnology Co., Ltd. | 99% |
| Crocetin | China Food and Drug Inspection Institute | 99% |
| Crocin III | Sichuan Pushide Biotechnology Co., Ltd. | 99% |
| Paroxetine Hydrochloride | Sino-US Tianjin SmithKline Pharmaceutical Co., Ltd. | 99% |
| β-Cyclodextrin | Tianjin Fuchen Chemical Reagent Factory | 99% |

Table S2 Experimental instruments and equipment

| Device Name | Model | Manufacturer |
| --- | --- | --- |
| High-performance liquid chromatography (HPLC) | LC-20A | Shimadzu Corporation |
| Triple Quadrupole Liquid Chromatography Mass Spectrometry(LC/MS) | LC-MS 2020 | Shimadzu Corporation |
| Glass capillary sampling tube | 10UL | Beijing Mingtai Jiaxin Technology Co., Ltd. |
| Vortex mixer | XH-C | Shanghai Dalo Scientific Instrument Co., Ltd. |
| Low-temperature high-speed centrifuge | Thermo Sorvall ST 16R | Thermo Fisher Scientific Inc. |
| High-throughput tissue grinder | CBGT-48 | Shanghai Cebo Biotechnology Development Center |

1. **Anxiolytic-like depression pharmacodynamic evaluation**

**1.1 Solution preparation**

Preparation of low-dose, medium-dose, and high-dose solutions of crocin I. The *β*-cyclodextrin solution containing 0.45% was prepared by weighing 1.8g of *β*-cyclodextrin, adding it to 200mL in double steaming water, and heating and stirring at 60℃. Weigh 90 mg of crocin I and dilute to volume with 0.45% β-cyclodextrin solution to prepare a high-dose solution of crocin I containing 1.8 mg/mL. Take 20 mL of the high-dose solution and dilute it 2 times with 0.45% β-cyclodextrin solution to obtain a medium-dose solution of crocin I. Take 15 mL of the medium-dose solution and continue to dilute it 2 times to obtain a low-dose solution of crocin I. Store in a brown bottle in the refrigerator at 4℃.

**1.2 Morris water maze**

The water maze round pool was divided into Ⅰ, Ⅱ, Ⅲ, and Ⅳ quadrants. Put it into the platform to calibrate the camera area and platform. Add water to the pool until it passes the platform 1cm or so, pour in black ink, and stir well. Until the aqueous solution is dyed black, the position of the platform can be covered. Put each group of SD rats head down along the pool wall into the water. The position was placed once along the Ⅰ, Ⅱ, Ⅲ, and Ⅳ quadrants respectively, and the time of finding the platform within 60 seconds was recorded. If the platform is not found within 60 seconds, the rats must be guided to the platform for 10 seconds to enhance their memory. Each rat is trained once a day in each quadrant for 5 consecutive days. After 5 days, the platform was removed, the animals were put into the pool from the original platform, and the number of rats crossing the target quadrant within 60 seconds was recorded.

**1.3 Sucrose preference test（SPT）**

1% sucrose aqueous solution was used in the experiment. The sweet food preference of healthy rats judged the degree of depression. Three days before the experiment, the animals in each group were fed separately and other factors such as social environment were excluded. One day before the experiment, the rats in each group were given sugar water adaptation. On the day of the test, two bottles containing the same volume of 1% sucrose solution and ordinary water were put into cages, weighed before and after drinking water, and the liquid consumption in the two bottles within 24 hours was calculated.

**1.4 Histopathological analysis of hippocampus and cortex in rats**

After the behavioral evaluation of the rats, the rats were anesthetized and decapitated to take the brain. The brain tissue was fixed with 4% paraformaldehyde for 24 hours. Rinse with normal saline to remove the residual fixative. Dehydration with 30% sucrose solution. It was embedded with an OTC embedding agent, and after it was completely frozen, the shape was trimmed and the sections were frozen. According to the localization map of rat brain sections, the target locations of the hippocampus and prefrontal cortex of rats were selected and sectioned continuously in the coronal plane with a thickness of 3 µm. Stick the slides with adhesive slides and bake the slides in an oven at 37 ℃ for half an hour. The slices were stained with methyl violet dye and rinsed with distilled water. Nissl Differentiation was used to differentiate for 5 s and most of the staining solution was removed. The slides were placed successively in different concentrations of ethanol and gradient dehydration (0%, 50%, 75%, and 95% ethanol, the section and background color should be observed under a microscope during dehydration). After dehydration, the sections were sealed with transparent and neutral gum in anhydrous ethanol Ⅰ, Ⅱ, anhydrous ethanol-xylene (1:1), xylene Ⅰ, and Ⅱ solution, respectively, and observed under a microscope.

**2. Pharmacokinetic analysis**

**2.2 Sample processing and analysis**

（1）Preparation of internal standard solution

Accurately weigh 1.0 mg of crocin III standard, add methanol to make up to 100 mL, and prepare a 0.01 mg/mL internal standard stock solution. The stock solution was further diluted to prepare 100 mL of internal standard solution with a concentration of 0.25 μg/mL for later use.

（2）Standard solution preparation

Accurately weigh 1.0 mg of crocetin, add methanol to make up to 100 mL, and prepare a standard stock solution with a concentration of 0.01 mg/mL. The stock solution was diluted to prepare a series of crocetin standard solutions with concentrations of 0.03125 μg/mL，0.0625 μg/mL，0.125 μg/mL，0.25 μg/mL，0.5 μg/mL，1 μg/mL.

（4）Standard solution treatment

Take 400 μL of rat blank plasma and add an equal volume of 400 μL of methanol. Then, 100 μL of the internal standard solution was added, vortexed for 3 min, 12000 rpm, 4°C, centrifuged for 10 min, and the supernatant was taken for later use.

**2.3 HPLC analysis**

Take 0.2 mL of plasma, add 2 times the amount of methanol, and vortex. Centrifuge at 5000 rpm for 10 min and take the supernatant. 10 μL of 1 μg/mL internal standard crocin III was added to the supernatant, and HPLC content was measured. The detection conditions adopt low-pressure gradient elution, and the mobile phase is a methanol-water system. The flow rate was 1 min/mL, the detection wavelength was 254 nm, the column temperature was 30°C, and the injection volume was 20 μL.

Crocin I concentration in plasma was determined by HPLC using a Shimadzu LC-2030 plus separation module coupled with a dual absorbance detector (Shimadzu, Milford, NS, Malaysia). Crocin I was separated on a Shim-pack GIST C18 column (250 mm, i.d., 4.6 μm ), with an ultra-column inline filter. A gradient solvent system consisting of methanol with aqueous is used as the mobile phase at a flow rate of 1 mL/min. Set the column oven temperature to 30 degrees, the running time to 40 minutes, and the injection volume to 20 μL. Crocin I was detected at a wavelength of 254 nm. The working standard solution for HPLC analysis was prepared by serial dilution of mobile phase methanol, with concentrations of 1, 0.5, 0.25, 0.125, 0.0625, and 0.03125 μg/mL.

Table S3 liquid chromatographic conditions of Crocin I, Ⅱ and Crocin Acid

| Time | Mobile phase (%) | | Velocity of flow | Sample size | Column oven |
| --- | --- | --- | --- | --- | --- |
| （min） | A（Methanol） | B（Water） | （mL/min） | （μL） | (℃) |
| 0 | 45 | 55 | 1 | 20 | 30 |
| 30 | 90 | 10 |
| 40 | 80 | 20 |

**2.4 Methodological investigation**

2.4.1 Precision

A low-dose, medium-dose, and high-dose plasma sample was taken 30 minutes after intragastric administration. Each sample was measured 6 times under the same conditions, and the precision and accuracy were calculated. The relative standard deviation (RSD) is less than 15%. The accuracy (RE) is within the range of 85-115%.

2.4.3 Stability

Plasma samples were collected 30 minutes after administration (n=6). The stability of the samples was calculated within one week at -20°C.

2.4.3 Limit of quantitation（LOQ）

Take 6 concentrations of standard solution for detection and draw a standard curve. Calculate the concentration of crocetin when the signal-to-noise ratio is equal to 10, which is the detection limit.

2.3.4 Sample Recovery Rate

Six portions of blank plasma from rats were collected, 3 of which were added with 50 μL of standard crocetin, and the other 3 were added with an equal volume of methanol. Calculate the concentration of crocetin in the spiked and unspiked solutions respectively. The recovery rate was calculated by the ratio of the concentration difference between the spiked and unspecified crocetin to the simple determination of the same concentration and volume of the internal standard.

**Figure S1 Number of neurons in specific areas of DG, CA1, and CA3**

**
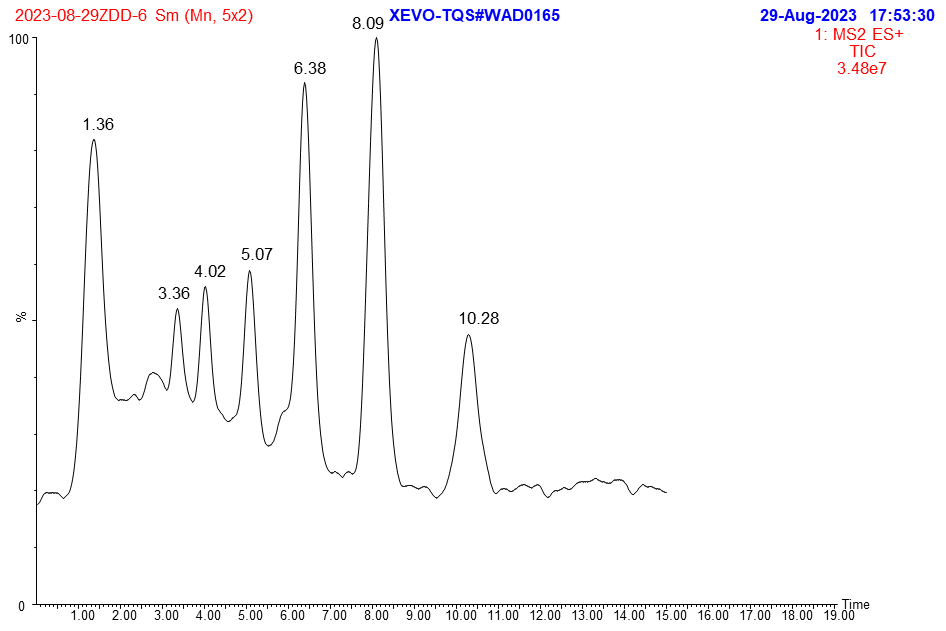
**

**Figure S2 Total ion current of crocin I acid hydrolysis**


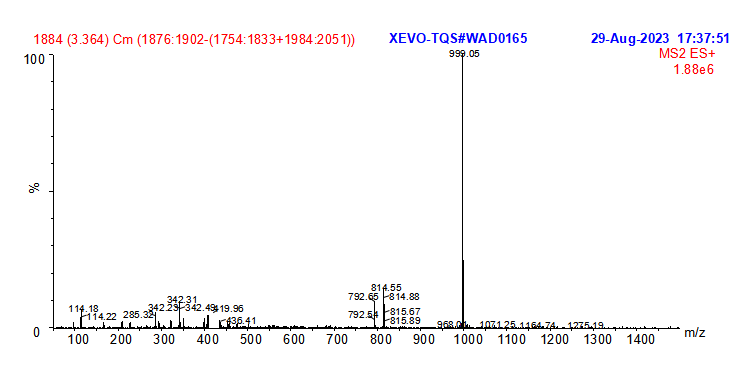


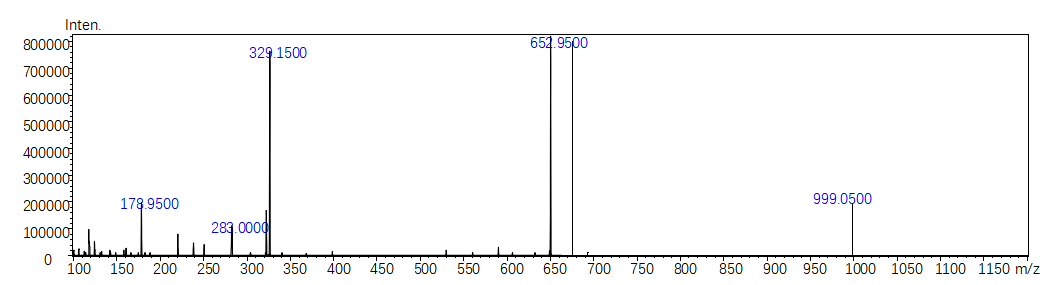


**Figure S3 Primary and secondary mass spectra of crocin I**


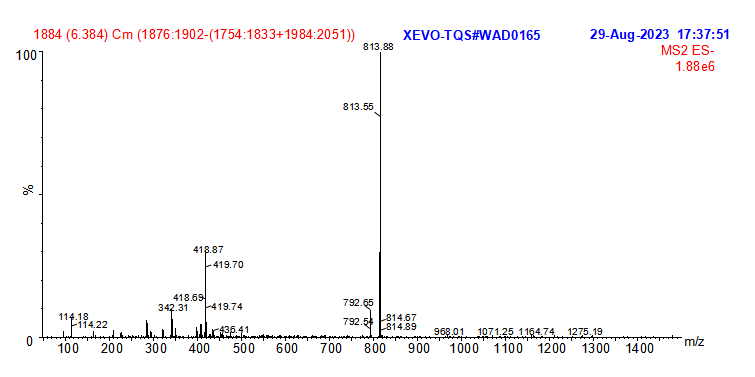


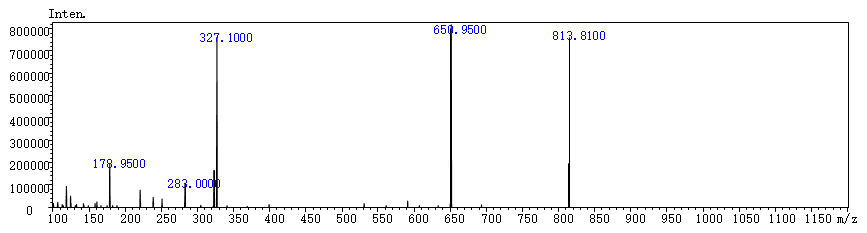


Fig. S4 Primary and secondary mass spectrometry of metabolites 1(Crocin Ⅱ)


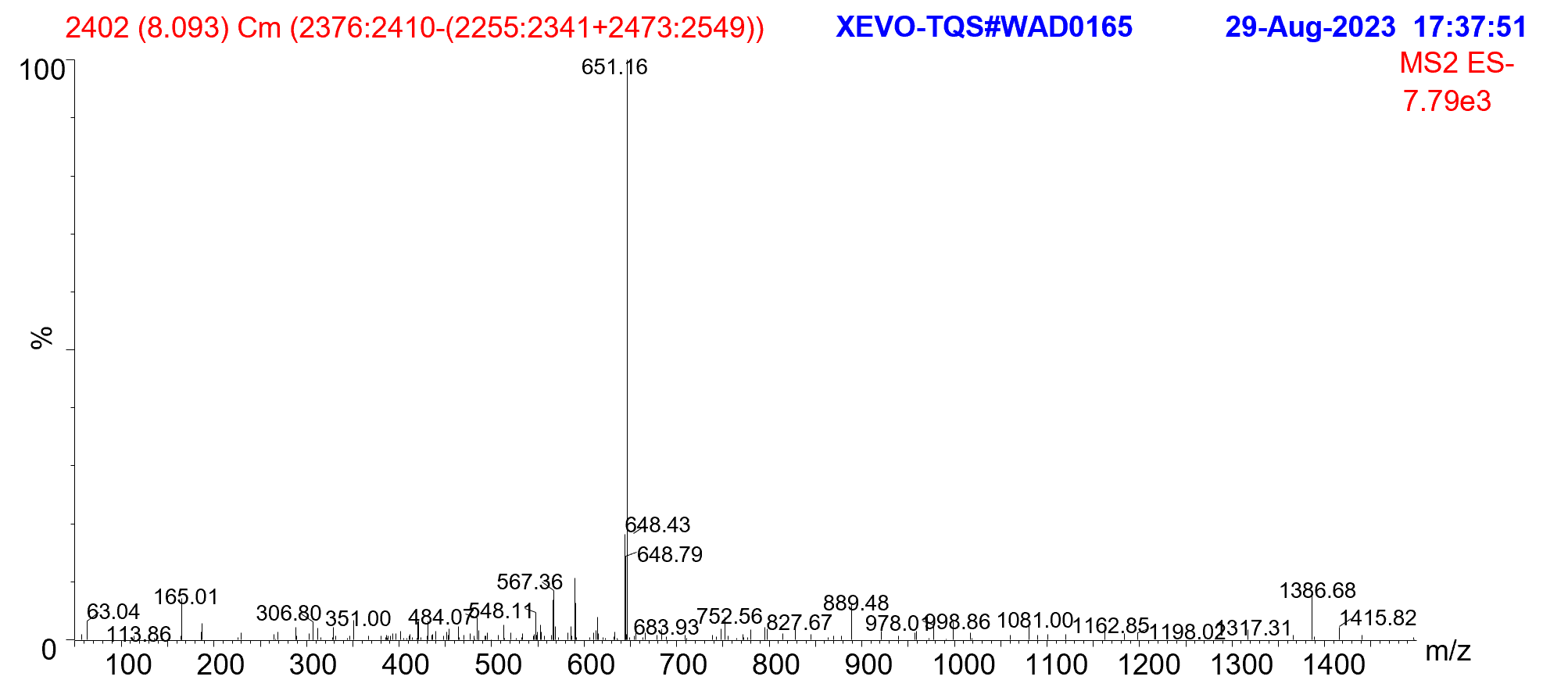

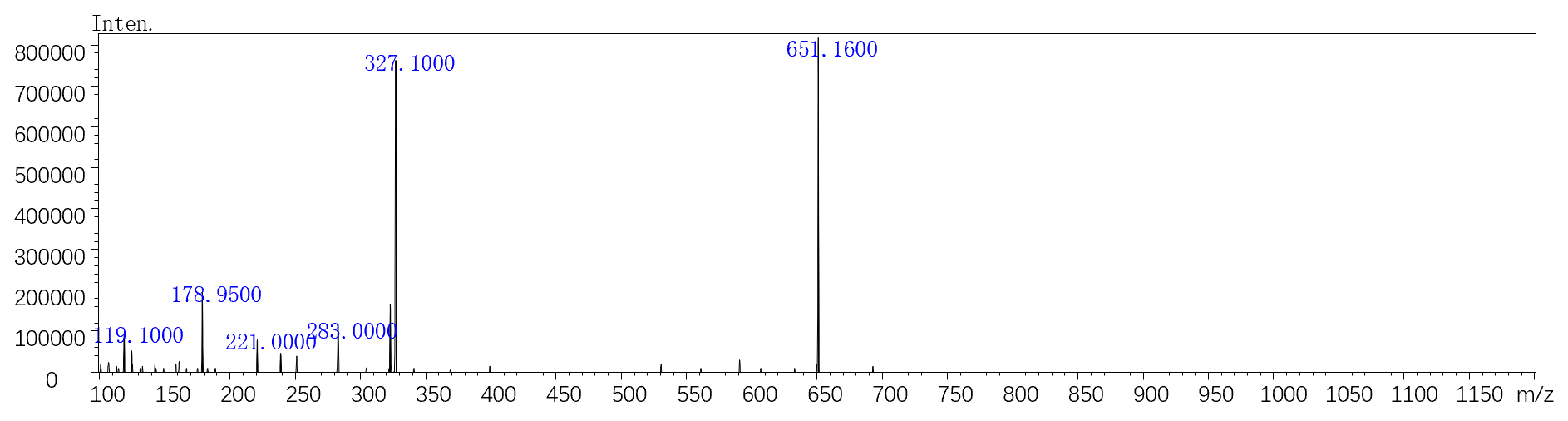


Fig. S5 Primary and secondary mass spectrometry of metabolites 2(Crocin Ⅳ)


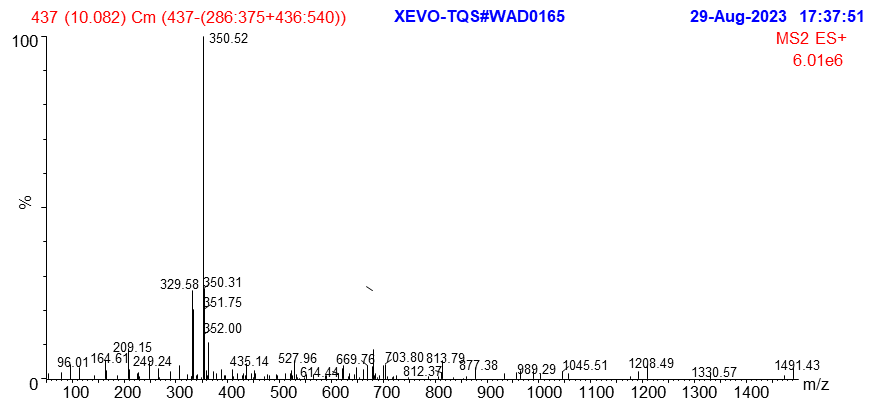


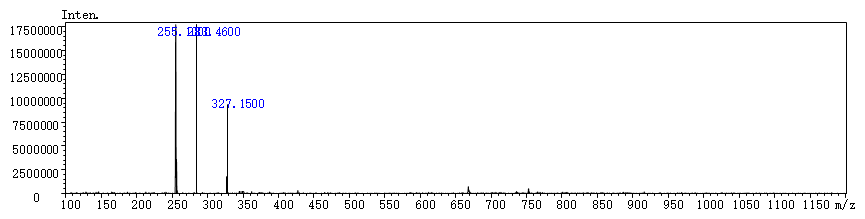


Fig. S6 Primary and secondary mass spectrometry of metabolites 3(Crocetin)

Fig. S7 Variation curve area with concentration. A. crocetin B. Crocin Ⅲ

Figure S8 AgNPs particle size distribution results
